# The Mitochondria-Targeted Methylglyoxal Sequestering Compound, MitoGamide, Is Cardioprotective in the Diabetic Heart

**DOI:** 10.1007/s10557-019-06914-9

**Published:** 2019-10-25

**Authors:** Mitchel Tate, Gavin C. Higgins, Miles J. De Blasio, Runa Lindblom, Darnel Prakoso, Minh Deo, Helen Kiriazis, Min Park, Carlos D. Baeza-Garza, Stuart T. Caldwell, Richard C. Hartley, Thomas Krieg, Michael P. Murphy, Melinda T. Coughlan, Rebecca H. Ritchie

**Affiliations:** 1 Heart Failure Pharmacology, Baker Heart and Diabetes Institute, Melbourne, VIC Australia; 2 Department of Diabetes, Monash University, Melbourne, VIC Australia; 3 JDRF Danielle Alberti Memorial Centre for Diabetic Complications, Diabetic Complications Division, Baker Heart and Diabetes Institute, Melbourne, Australia; 4 Experimental Cardiology, Baker Heart and Diabetes Institute, Melbourne, VIC Australia; 5 WestCHEM School of Chemistry, University of Glasgow, Glasgow, G12 18QQ UK; 6 Department of Medicine, University of Cambridge, Cambridge Biomedical Campus, Cambridge, UK; 7 MRC Mitochondrial Biology Unit, University of Cambridge, Cambridge Biomedical Campus, Cambridge, CB2 0XY UK

**Keywords:** Diabetes, Heart, Diabetic cardiomyopathy, Methylglyoxal

## Abstract

**Purpose:**

Methylglyoxal, a by-product of glycolysis and a precursor in the formation of advanced glycation end-products, is significantly elevated in the diabetic myocardium. Therefore, we sought to investigate the mitochondria-targeted methylglyoxal scavenger, MitoGamide, in an experimental model of spontaneous diabetic cardiomyopathy.

**Methods:**

Male 6-week-old Akita or wild type mice received daily oral gavage of MitoGamide or vehicle for 10 weeks. Several morphological and systemic parameters were assessed, as well as cardiac function by echocardiography.

**Results:**

Akita mice were smaller in size than wild type counterparts in terms of body weight and tibial length. Akita mice exhibited elevated blood glucose and glycated haemoglobin. Total heart and individual ventricles were all smaller in Akita mice. None of the aforementioned parameters was impacted by MitoGamide treatment. Echocardiographic analysis confirmed that cardiac dimensions were smaller in Akita hearts. Diastolic dysfunction was evident in Akita mice, and notably, MitoGamide treatment preferentially improved several of these markers, including e′/a′ ratio and E/e′ ratio.

**Conclusions:**

Our findings suggest that MitoGamide, a novel mitochondria-targeted approach, offers cardioprotection in experimental diabetes and therefore may offer therapeutic potential for the treatment of cardiomyopathy in patients with diabetes.

**Electronic supplementary material:**

The online version of this article (10.1007/s10557-019-06914-9) contains supplementary material, which is available to authorized users.

## Introduction

Diabetes is a well-known risk factor for the development of cardiovascular diseases, pertinent since the global incidence of diabetes is set to exceed 642 million by 2040 [1]. Cardiovascular disease is arguably the most important complication of diabetes, increasing heart failure risk 2.4-fold in diabetic men and 5-fold in diabetic women [2, 3], but also accounting for the majority of healthcare costs and significantly reducing life expectancy. In one study, the 1-year mortality of heart failure was 30% in people with diabetes, about 1.5-fold greater than in those without diabetes [4]. Diabetic heart disease exists across a range of aetiologies, including coronary heart disease, diabetic cardiomyopathy and heart failure. Diabetes can expedite or even initiate changes in each disease scenario. For example, diabetes accelerates the progression of atherosclerosis in coronary arteries, exacerbates small vessel disease leading to increased cardiac load, compromised cardiac performance and eventual heart failure, and promotes detrimental cardiac remodelling characteristic of diabetic cardiomyopathy, including cardiomyocyte hypertrophy, elevated interstitial fibrosis and cardiomyocyte apoptosis [5].

**Fig. 1:**
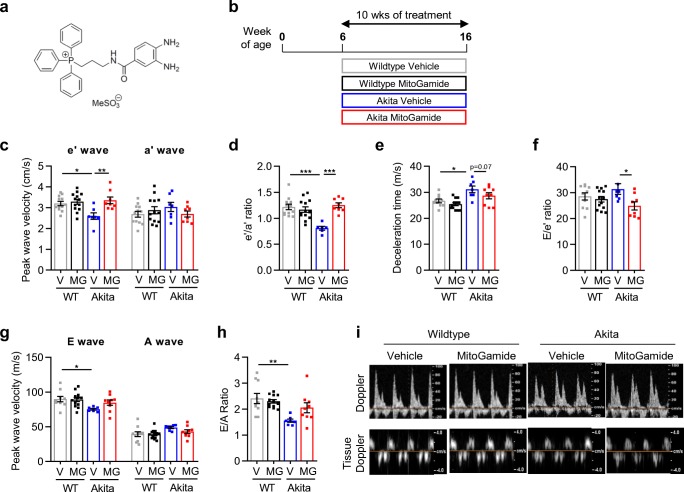
MitoGamide treatment attenuates LV diastolic dysfunction in Akita mice. **a** The structure of MitoGamide. **b** Overview of experimental protocol. Tissue Doppler echocardiography was used to derive **c** peak e′ and peak a′ velocity, and **d** e′/a′ ratio. Doppler echocardiography was used to derive **e** deceleration time. **f** E/e′ ratio **g** peak E and peak A wave velocity, and **h** E/A ratio. **i** Representative images of Doppler and tissue Doppler echocardiography. Data are presented as mean ± SEM. *n* = 8–13 per group. *P < 0.05, **P < 0.01, ****P < 0.0001. Two-way ANOVA followed by Tukey’s post hoc test. V, vehicle; MG, MitoGamide; WT, wild type

The heart is the most metabolically active organ in the body and possesses the highest amount of mitochondria, the powerhouses of the cell [6]. The high myocardial mitochondria content is absolutely critical in order to provide the tremendous amount of energy needed to maintain cardiac contraction and relaxation. In fact, despite the heart accounting for only 0.5% of body weight, it is responsible for 8% of ATP consumption [6]. Given that tissues with a greater metabolic demand are more susceptible to chronic complications, mitochondrial impairment has been implicated in the pathophysiology of diabetic heart disease [7]. Methylglyoxal, a by-product of glycolysis and a reactive carbonyl species, is significantly elevated in diabetes and reacts with arginine and lysine residues to form irreversible carbonyl adducts [8]. Importantly, mitochondrial proteins are major targets of dicarbonyl glycation and are associated with increased reactive oxidative species formation and subsequent cardiac damage [9]. We hypothesise that the metabolic derangements indicative of diabetes, and subsequent dicarbonyl glycation, play a role in the development of diabetic cardiomyopathy. MitoGamide is an amide analogue of MitoG [10], which has been used to assess the accumulation of glyoxal and methylglyoxal in the mitochondria of the Akita mouse model of diabetes in vivo [10]. The two molecules share the methylglyoxal-scavenging diaminoaryl group and the mitochondria-targeting triphenylphosphonium moiety, but MitoGamide incorporates an amide link to facilitate synthesis and limit autooxidation. Therefore, in the present study we sought to investigate the cardioprotective potential of the novel mitochondria-targeted methylglyoxal scavenger, MitoGamide (Fig. 1a), in the setting of experimental diabetic cardiomyopathy.

## Methods

### Animals

All activities involving the use of animals for research were approved by the Alfred Medical Research Education Precinct (AMREP) Animal Ethics Committee and were conducted according to guidelines of the National Health and Medical Research Council of Australia for animal experimentation. For all experiments, we have included flow charts for the reporting of animal use and analysis in preclinical studies (Supplementary Fig. 1). The main aim of this study was to investigate the impact of MitoGamide treatment on cardiac function in an experimental model of diabetic cardiomyopathy. Accordingly, our primary endpoint was impact of MitoGamide treatment on e′/a′ ratio and E/A ratio, markers of LV diastolic function. Diabetic Akita mice (C57BL/6J-Ins2Akita; heterozygous for the mutation) and their wild type (WT) counterparts were purchased from the Jackson Laboratory, bred at the AMREP Animal Centre and maintained under a 12-h light/dark cycle. At 6 weeks of age, male littermates of both genotypes were assigned to receive either vehicle (10% ethanol in water), or MitoGamide (10 mg/kg) by daily oral gavage. Saphenous vein nonfasted blood glucose measurements (“High” measurements recorded at 33.3 mmol/L; ACCU-CHEK glucometer, Roche) and body weights were recorded on a weekly basis as part of animal monitoring. Glycated haemoglobin (HbA_1c_) was measured 1 week prior to study end using the Cobas B 101 system (Roche). Plasma insulin levels were measured using Mouse Ultrasensitive Insulin ELISA kit, as per manufacturer’s instructions (80-INSMSU-E01, ALPCO). At study end, animals received an overdose of sodium pentobarbital (80 mg/kg i.p.) prior to rapid excision and collection of hearts.

### Echocardiography

Echocardiography was performed in anaesthetised mice (ketamine/xylazine/atropine: 100/10/0.96 mg/kg i.p. at study endpoint utilising a Philips iE33 ultrasound machine with 15-MHz linear (M-mode) and 12-MHz sector (Doppler) transducer. LV posterior wall (PWd) thickness, LV chamber dimensions and fractional shortening were assessed from M-mode imaging. LV filling was assessed using transmitral Doppler flow; the ratio of early (E) and atrial (A) blood flow velocities (E/A ratio) and E-wave deceleration time were measured. Tissue Doppler echocardiography was used to assess the ratio of e′ velocity and a′ velocity (e′/a′ ratio).

### Statistical Analysis

Data were analysed with GraphPad Prism 8.01 statistical software package. All data are presented as mean ± standard error of the mean (SEM). Two-way analysis of variance (ANOVA) was used to detect main effects for genotype (WT vs Akita) and treatment (vehicle vs MitoGamide), followed by Tukey’s post hoc test to analyse the differences between individual experimental groups. Statistical significance was considered at *P* < 0.05.

## Results

### Characterisation of Diabetes and Cardiac Dimensions

Male Akita or wild type mice at 6 weeks of age received daily oral gavage of MitoGamide or vehicle for a duration of 10 weeks (Fig. 1b). At study end, body weight and tibial length were smaller in diabetic mice compared with wild type (Table 1), consistent with previous reports using this model [11]. Blood glucose and glycated haemoglobin levels were significantly elevated in Akita mice (Table 1). Plasma insulin levels were reduced in Akita mice compared with wild type mice (Table 1). MitoGamide treatment had no effect compared with vehicle treatment on the aforementioned measures. Heart weight normalised to tibial length was significantly reduced in the Akita vehicle-treated mice compared with wild type vehicle-treated mice (Table 1). When LV weight and RV weight were considered independently, they mirrored the results seen in total heart weight (Table 1).

**Table 1:** Systemic characteristics, organ morphology and echocardiographic analysis of systolic heart function in anaesthetised wild type and Akita mice treated with MitoGamide or vehicle

	Wild type		Akita	
	Vehicle	MitoGamide	Vehicle	MitoGamide
Systemic characteristics/organ morphology				
*n*	14	15	14	12
Body weight (g)	29.9 ± 1.2	28.5 ± 0.5	22.8 ± 0.9****	23.7 ± 0.7**
Tibial length (mm)	17.3 ± 0.1	17.1 ± 0.2	16.7 ± 0.1*	16.4 ± 0.1**
Blood glucose (mmol/L)	10.8 ± 0.6	11.7 ± 0.7	33.0 ± 0.0****	32.6 ± 0.4****
HbA_1c_ (%)	4.60 ± 0.17	4.83 ± 0.16	13.1 ± 0.35****	13.3 ± 0.26****
Plasma insulin (ng/mL)	0.89 ± 0.04	0.92 ± 0.07	0.75 ± 0.01*	0.78 ± 0.02
Heart weight/tibial length (mg/mm)	9.19 ± 0.42	8.26 ± 0.33	7.02 ± 0.13****	7.72 ± 0.33
LV/tibial length (mg/mm)	6.18 ± 0.27	5.57 ± 0.19	4.50 ± 0.10****	5.05 ± 0.16
RV/tibial length (mg/mm)	1.64 ± 0.09	1.54 ± 0.08	1.25 ± 0.06**	1.30 ± 0.07
LV function				
*n*	12	13	9	10
Heart rate (bpm)	399 ± 17	418 ± 11	381 ± 10	385 ± 15
Ex-LVEDD (mm)	5.75 ± 0.11	5.51 ± 0.09	5.19 ± 0.05**	5.31 ± 0.09
AWd (mm)	0.67 ± 0.02	0.67 ± 0.01	0.60 ± 0.02*	0.61 ± 0.01*
LVEDD (mm)	4.40 ± 0.12	4.19 ± 0.10	4.01 ± 0.08	4.09 ± 0.12
PWd (mm)	0.68 ± 0.02	0.68 ± 0.02	0.60 ± 0.01***	0.63 ± 0.01
LVESD (mm)	3.15 ± 0.08	2.96 ± 0.10	2.55 ± 0.09***	2.58 ± 0.09*
Fractional shortening (%)	28.3 ± 0.5	29.5 ± 1.0	36.5 ± 1.6****	37.0 ± 0.8****
Estimated LV mass (mg)	112 ± 5	101 ± 4	81 ± 2***	87 ± 3
Estimated LV mass/BW (mg/g)	3.76 ± 0.19	3.53 ± 0.10	3.68 ± 0.14	3.74 ± 0.21
Estimated LV mass/TL (mg/mm)	6.47 ± 0.29	5.90 ± 0.24	4.84 ± 0.13***	5.29 ± 0.19

Data are presented as mean ± SEM and analysed by two-way ANOVA followed by Tukey’s post hoc test. *P < 0.05, **P < 0.01, ***P < 0.001, ****P < 0.0001 vs corresponding wildtype. *Ex-LVEDD*, external LV end diastolic dimension; *Awd*, anterior wall diastolic thickness; *LVEDD*, LV end diastolic dimension; *PWd*, posterior wall diastolic thickness; *LVESD*, LV end systolic dimension; *BW*, body weight; *TL*, tibial length

### Cardiac Function

Tissue Doppler imaging showed a significant decrease in peak e′ wave velocity in Akita vehicle-treated mice compared with wild-type vehicle-treated mice (Fig. 1c), an effect attenuated by MitoGamide treatment. There was no difference between groups in terms of peak a′ velocity (Fig. 1c). This translated into a significant decrease in e′/a′ ratio in the Akita vehicle-treated mice that was attenuated following MitoGamide treatment (Fig. 1d). There was a clear trend evident that MitoGamide treatment impacted the prolongation of deceleration time observed in vehicle-treated Akita mice (*P* = 0.07, Fig. 1e). E/e′ ratio was reduced in MitoGamide-treated Akita mice compared with Akita vehicle mice (Fig. 1f). Doppler echocardiographic assessment of LV diastolic function indicated a significant decrease in E-wave velocity in Akita mice (Fig. 1g). However, there was no difference between groups in terms of peak A wave velocity (Fig. 1g). This translated to a significant reduction in E/A ratio, indicative of LV diastolic dysfunction (Fig. 1h); this impairment was not evident in MitoGamide-treated Akita mice. Representative images of tissue Doppler imaging and Doppler imaging are provided in Fig. 1i. These findings reveal that MitoGamide treatment in diabetic mice protects against the development of impaired ventricular relaxation.

LV M-mode echocardiographic structure and systolic function were significantly different between wild type and Akita mice, indicated by a reduction in LV end-systolic and end-diastolic dimensions, including Ex-LVEDD, AWd, PWd and LVESD (Table 1). Fractional shortening was significantly elevated in Akita mice compared with age-matched wild type mice. Importantly, MitoGamide treatment exerted no impact on echocardiographic parameters of LV structure and M-mode-derived systolic function, and heart rate remained unchanged, between all groups for echocardiographic measurements (Table 1).

## Discussion

The key finding of this study is the novel compound MitoGamide, an amide variant of MitoG [10] and mitochondria-targeted methylglyoxal scavenger, offers cardioprotection in the Akita mouse model of spontaneous experimental diabetes. MitoGamide treatment exhibited no impact on the underlying diabetes phenotype; however, MitoGamide preferentially improved several markers of LV diastolic dysfunction in diabetic mice. Given the well-established link between the increased global burden of diabetes and an increased risk of clinical heart failure [2], identification of new pharmacological treatment strategies designed specifically to target the underlying metabolic perturbations in disease pathogenesis is particularly timely [3].

Diabetes is a complex metabolic disease characterised primarily by hyperglycaemia but with many other interacting factors that lead to a broad range of complications, including diabetic heart disease. The rationale for this study was based on reports that methylglyoxal levels are elevated in diabetes [8, 12], as the enzyme responsible for the removal of methylglyoxal, glyoxalase 1, becomes saturated. Accumulating levels of methylglyoxal provide an important precursor for the nonenzymatic glycation of proteins, affecting the structure and function of proteins and ultimately affecting intracellular events [10]. Mitochondrial proteins are major targets of dicarbonyl glycation and are associated with increased reactive oxidation species formation and subsequent cardiac damage [9]. Methylglyoxal affects several cellular functions such as insulin signalling, mitochondrial respiration and glycolysis, whilst high-dose methylglyoxal therapy has been highlighted as a potential therapeutic option in cancer settings due to its cytotoxic effects [13]. Importantly, several mitochondria-targeted antioxidants confer beneficial improvements on cardiac function, although, to our knowledge, this is the first study to investigate the effect of a mitochondrial-targeted approach to reduce methylglyoxal levels in the diabetic heart.

To specifically address the potential for selectively targeting mitochondrial methylglyoxal in the diabetic heart, we chose to employ an established genetic model of maturity onset diabetes of the young (MODY)4 and insulin insufficiency using the Ins2^WT/C96Y^ Akita mouse model. Although this model may not be as clinically relevant as one that mimics the more prevalent type 2 diabetes, utilising the Akita mouse model avoids the additional confounding factors of obesity and impaired leptin signalling evident in the most widely accepted mouse model of type 2 diabetes that manifests detectable cardiac dysfunction, the spontaneously diabetic *db/db* mouse. Importantly, the Akita mouse model shares common structural and functional features of clinical diabetic cardiomyopathy and was therefore an appropriate choice for this study. Although the pathogenesis of type 1 and type 2 diabetes are distinct at the systemic level, the changes that occur in the myocardium, and in particular the cardiac mitochondria, in both forms of diabetes, share numerous similarities [7].

The early stages of diabetic cardiomyopathy are commonly characterised by LV diastolic dysfunction and ventricular hypertrophy, and in later stages by LV systolic dysfunction progressing to decompensated heart failure [3, 5]. Consistent with previous studies [14], our data confirm the presence of LV diastolic dysfunction at 16 weeks of age in Akita mice. Evaluation of both Doppler flow and tissue Doppler echocardiography revealed that 16 weeks of hyperglycaemia conferred a reduction in e′/a′ ratio and E/A ratio, and a prolongation of deceleration time (Fig. 1). Importantly, myocardial function of diabetic mice treated with MitoGamide for the final 10 weeks of the study exhibited significant improvements in e′/a′ ratio and E/e′ ratio and a non-significant improvement in E/A ratio (*P* = 0.16, Fig. 1), indicating that MitoGamide offers cardioprotection in this experimental model of diabetic cardiomyopathy. These reports are consistent with previous reports that overexpression of glyoxalase 1, the enzyme responsible for removing accumulating levels of methylglyoxal, in a type 1 model of diabetic cardiomyopathy, delayed and limited impairment in cardiac function [14]. At the time point in which cardiac function was assessed in our study, it is important to note that LV end-systolic and end-diastolic dimensions were smaller in the Akita mice and there was a significantly higher fractional shortening (Table 1). These findings are consistent with other studies using this model [15] and likely explained by the genetic nature of the model, where Akita mice exhibit smaller body weight, tibial length, heart and LV size when normalised to tibial length (Table 1). The phenotype of the Akita mouse hence precludes any inference regarding the presence or absence of LV systolic dysfunction in this model; however, MitoGamide treatment did not affect any of these parameters (Table 1).

Future directions, based on the findings outlined in this short communication, will likely include substantiating the role of MitoGamide in the more clinically relevant setting of type 2 diabetes. Emphasis can then be placed on understanding the role, if any, of MitoGamide on mitochondrial function and oxidative stress. Furthermore, the current studies only describe the ability of MitoGamide to prevent the development of cardiac dysfunction, whereas future studies will determine the ability of MitoGamide to protect against established cardiac dysfunction, the most likely clinical scenario. This will include the use of serial echocardiography. Indeed, it is likely any novel treatment option that has efficacy in terms of cardiac function will supplement (not replace) current therapeutic options in the management of disease. Further, one limitation of the current study was that it only included male mice. Future studies will determine if therapeutic intervention is effective in both male and female mice.

Taken together, our data indicate that MitoGamide treatment confers cardioprotection in an experimental model of diabetic cardiomyopathy, and although further work is clearly required to elucidate the underlying mechanisms, our findings highlight a novel mitochondria-targeted approach that may prevent the onset, or slow the progression, of diabetic cardiomyopathy.

## Electronic supplementary material

ESM 1 (PPTX 91 kb)

## Abbreviations

AWd: anterior wall diastolic thickness
AMREP: Alfred Medical Research Education Precinct
ANOVA: two-way analysis of variance
BW: body weight
Ex-LVEDD: external LV end diastolic dimension
LVESD: LV end systolic dimension
HbA_1c_: Glycated haemoglobin
LV: left ventricular
LVEDD: LV end diastolic dimension
PWd: posterior wall diastolic thickness
SEM: standard error of the mean
TL: tibial length
WT: wild type
