# Supplementary material for: The Mitochondria-Targeted Methylglyoxal Sequestering Compound, MitoGamide, Is Cardioprotective in the Diabetic Heart
Source: Cardiovasc Drugs Ther. 2019 Oct 25;33(6):669–74. doi: 10.1007/s10557-019-06914-9 (PMC6994445; doi:10.1007/s10557-019-06914-9)
Supplement: Supplementary file 1 — (PPTX 91 kb) [file 10557_2019_6914_MOESM1_ESM.pptx]

## Slide 1
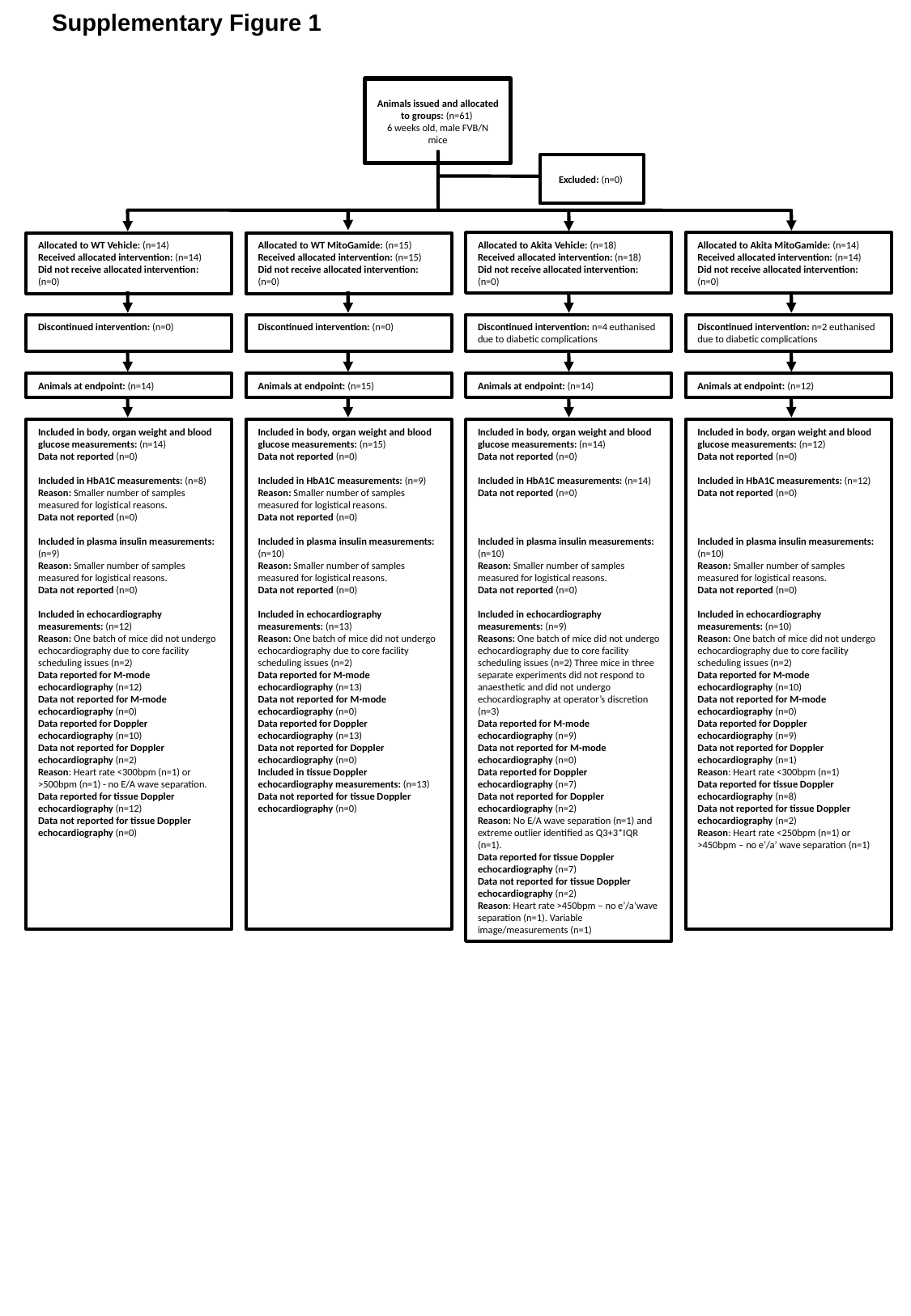

Supplementary Figure 1
Animals issued and allocated to groups: (n=61)
6 weeks old, male FVB/N mice
Excluded: (n=0)
Allocated to Akita Vehicle: (n=18)
Received allocated intervention: (n=18)
Did not receive allocated intervention: (n=0)
Allocated to Akita MitoGamide: (n=14)
Received allocated intervention: (n=14)
Did not receive allocated intervention: (n=0)
Allocated to WT Vehicle: (n=14)
Received allocated intervention: (n=14)
Did not receive allocated intervention: (n=0)
Allocated to WT MitoGamide: (n=15)
Received allocated intervention: (n=15)
Did not receive allocated intervention: (n=0)
Discontinued intervention: (n=0)
Discontinued intervention: (n=0)
Discontinued intervention: n=4 euthanised due to diabetic complications
Discontinued intervention: n=2 euthanised due to diabetic complications
Animals at endpoint: (n=14)
Animals at endpoint: (n=15)
Animals at endpoint: (n=14)
Animals at endpoint: (n=12)
Included in body, organ weight and blood glucose measurements: (n=14)
Data not reported (n=0)
Included in HbA1C measurements: (n=8)
Reason: Smaller number of samples measured for logistical reasons.
Data not reported (n=0)
Included in plasma insulin measurements: (n=9)
Reason: Smaller number of samples measured for logistical reasons.
Data not reported (n=0)
Included in echocardiography measurements: (n=12)
Reason: One batch of mice did not undergo echocardiography due to core facility scheduling issues (n=2)
Data reported for M-mode echocardiography (n=12)
Data not reported for M-mode echocardiography (n=0)
Data reported for Doppler echocardiography (n=10)
Data not reported for Doppler echocardiography (n=2)
Reason: Heart rate <300bpm (n=1) or >500bpm (n=1) - no E/A wave separation.
Data reported for tissue Doppler echocardiography (n=12)
Data not reported for tissue Doppler echocardiography (n=0)
Included in body, organ weight and blood glucose measurements: (n=15)
Data not reported (n=0)
Included in HbA1C measurements: (n=9)
Reason: Smaller number of samples measured for logistical reasons.
Data not reported (n=0)
Included in plasma insulin measurements: (n=10)
Reason: Smaller number of samples measured for logistical reasons.
Data not reported (n=0)
Included in echocardiography measurements: (n=13)
Reason: One batch of mice did not undergo echocardiography due to core facility scheduling issues (n=2)
Data reported for M-mode echocardiography (n=13)
Data not reported for M-mode echocardiography (n=0)
Data reported for Doppler echocardiography (n=13)
Data not reported for Doppler echocardiography (n=0)
Included in tissue Doppler echocardiography measurements: (n=13)
Data not reported for tissue Doppler echocardiography (n=0)
Included in body, organ weight and blood glucose measurements: (n=14)
Data not reported (n=0)
Included in HbA1C measurements: (n=14)
Data not reported (n=0)
Included in plasma insulin measurements: (n=10)
Reason: Smaller number of samples measured for logistical reasons.
Data not reported (n=0)
Included in echocardiography measurements: (n=9)
Reasons: One batch of mice did not undergo echocardiography due to core facility scheduling issues (n=2) Three mice in three separate experiments did not respond to anaesthetic and did not undergo echocardiography at operator’s discretion (n=3)
Data reported for M-mode echocardiography (n=9)
Data not reported for M-mode echocardiography (n=0)
Data reported for Doppler echocardiography (n=7)
Data not reported for Doppler echocardiography (n=2)
Reason: No E/A wave separation (n=1) and extreme outlier identified as Q3+3*IQR (n=1).
Data reported for tissue Doppler echocardiography (n=7)
Data not reported for tissue Doppler echocardiography (n=2)
Reason: Heart rate >450bpm – no e’/a’wave separation (n=1). Variable image/measurements (n=1)
Included in body, organ weight and blood glucose measurements: (n=12)
Data not reported (n=0)
Included in HbA1C measurements: (n=12)
Data not reported (n=0)
Included in plasma insulin measurements: (n=10)
Reason: Smaller number of samples measured for logistical reasons.
Data not reported (n=0)
Included in echocardiography measurements: (n=10)
Reason: One batch of mice did not undergo echocardiography due to core facility scheduling issues (n=2)
Data reported for M-mode echocardiography (n=10)
Data not reported for M-mode echocardiography (n=0)
Data reported for Doppler echocardiography (n=9)
Data not reported for Doppler echocardiography (n=1)
Reason: Heart rate <300bpm (n=1)
Data reported for tissue Doppler echocardiography (n=8)
Data not reported for tissue Doppler echocardiography (n=2)
Reason: Heart rate <250bpm (n=1) or >450bpm – no e’/a’ wave separation (n=1)
